# Microsatellite markers for *Corybas* (Orchidaceae) species in New Zealand

**DOI:** 10.1002/aps3.1192

**Published:** 2018-11-09

**Authors:** Megan L. Van Etten, Carlos A. Lehnebach, Sofie M. Pearson, Alastair W. Robertson, Jennifer A. Tate

**Affiliations:** ^1^ School of Agriculture and Environment Massey University Palmerston North 4442 New Zealand; ^2^ Museum of New Zealand Te Papa Tongarewa Wellington 6011 New Zealand; ^3^ Institute of Fundamental Sciences Massey University Palmerston North 4442 New Zealand; ^4^ Present address: Penn State Scranton Dunmore Pennsylvania 18512 USA

**Keywords:** *Corybas*, *Corybas obscurus*, microsatellite, New Zealand, Orchidaceae, spider orchid

## Abstract

**Premise of the Study:**

Microsatellite markers were developed for New Zealand species of *Corybas* (Orchidaceae) to investigate population genetics and species delimitation.

**Methods and Results:**

From sequencing a total genomic DNA library (using Illumina MiSeq), we developed 22 microsatellite markers for *C. obscurus*. The di‐ and trinucleotide repeat loci were initially trialed on individuals representing seven *Corybas* taxa (*C*. “rimutaka,” *C. confusus*,* C. hypogaeus*,* C. macranthus*,* C. obscurus*,* C. trilobus*, and *C. walliae*) and had one to eight alleles per locus. Twelve polymorphic markers were further tested on six *Corybas* populations from three of the seven taxa (*C. obscurus*,* C*. “rimutaka,” and *C. trilobus*). Observed and expected heterozygosities ranged from 0–1 and 0–0.859, respectively. The utility of these 12 loci was further validated in five related *Corybas* species (*C. hypogaeus*,* C. obscurus*,* C. vitreus*,* C. walliae*, and *C*. “rimutaka”; 38 individuals) representing populations from across the North and South Islands. The average value for genetic diversity among populations (*F*_ST_) of 0.439 shows differentiation among species.

**Conclusions:**

These markers will be useful for future studies aimed at delimiting species boundaries and examining the genetic diversity of the New Zealand *Corybas* species.

*Corybas* Salisb. is a diverse genus of terrestrial orchids that includes ca. 150 species that are widespread across Australasia and Southeast Asia (Lyon, 2014). These orchids consist of a single leaf and flower, making the entire plant only a few centimeters tall and rather inconspicuous. Some species grow in sympatry and can also form dense and extensive intermingling clonal populations where hybridization may occur. In New Zealand, there are currently 21 accepted species of *Corybas* (Breitwieser et al., 2017). This number is likely to increase as there are several morphologically distinct entities known only by tag names that require genetic assessment to fully confirm their taxonomic status. This situation is not unique to New Zealand *Corybas*; a number of species aggregates have also been detected in Australian *Corybas* (Brown et al., 2008).

A recent study described five new species of *Corybas* endemic to New Zealand (Lehnebach et al., 2016) and identified at least two other morphologically distinct entities. One of the new species, *C. obscurus* Lehnebach, is considered “At Risk – Naturally Uncommon” in the list of Threatened and Uncommon Plants of New Zealand (de Lange et al., 2018) because it is restricted to a small area in the South Island. The undescribed entity *C*. “rimutaka” also occurs in this area, but chloroplast and nuclear sequence data were unable to discriminate this taxon from some samples of *C. obscurus* and the sympatric species *C. hypogaeus* (Colenso) Lehnebach (Lehnebach et al., 2016). These plants are all part of the *C. trilobus* (Hook. f.) Rchb. f. species aggregate. Floral characters are particularly variable within this species aggregate, and it is likely that several taxa are included under this name (St. George, 2008). A few of these nameless orchids are also considered “Threatened” or “At Risk” (de Lange et al., 2018).

Understanding the distribution of the morphological variation in New Zealand *Corybas* and determining taxonomic status is critical to ensure survival and suitable management of their populations. Genetic markers will aid in delimiting species boundaries and linking patterns of morphological and genetic variation. Therefore, we have developed a set of microsatellite markers that will be used in future studies to delimit species boundaries between closely related entities and detect gene flow between co‐occurring species.

## METHODS AND RESULTS

DNA was extracted from *C. obscurus* (WELT‐SP104152; Appendix 1) using a DNeasy Plant Mini Kit (QIAGEN, Hilden, Germany) with slight modifications of the manufacturer's protocol (0.5% β‐mercaptoethanol [BME] added to Buffer AP1; incubated at 65°C for 15 min; chilled on ice for 10 min). A DNA library was prepared using the Illumina TruSeq Library Preparation Kit (Illumina, San Diego, California, USA) following manufacturer's protocols. The indexed library was pooled with three other libraries (*Fuchsia excorticata* [Onagraceae; Van Etten et al., 2013], *Sophora microphylla* [Fabaceae; Van Etten et al., 2014], and *Korthalsella salicornioides* [Salicaceae; S. M. Pearson et al., unpublished]) in equal concentration and sequenced via Illumina MiSeq (Illumina) using 250‐bp paired‐end chemistry (New Zealand Genomics Limited, Palmerston North, New Zealand). The resulting 2.6 million sequences (991 million base pairs) were trimmed of low‐quality results using a 0.01 quality cut‐off in DynamicTrim in SolexaQA (Cox et al., 2010), and the remaining sequences were assembled using Velvet version 1.1 (Zerbino and Birney, 2008). The raw data were deposited in the National Center for Biotechnology Information (NCBI) Sequence Read Archive (SRA; accession SRP150798).

Plastid and mitochondrial sequences were removed by performing BLAST searches against related organellar sequences in GenBank (plastid: *Phalaenopsis aphrodite* Rchb. f. [Orchidaceae], NC_007499; mitochondria: *Tripsacum dactyloides* (L.) L. [Poaceae], NC_008362). Phobos (version 3.3.12; Mayer, 2010) was used to identify di‐ to hexanucleotide repeats with a length of ≥6 repeat units, resulting in 111,813 repeat regions. Primers were designed from regions containing a single uninterrupted repeat type in Geneious (Biomatters Ltd., Auckland, New Zealand) using Primer3 (Rozen and Skaletsky, 1999) with default settings except: product size = 100–300 bp; primer size = 17 (minimum)–19 (optimal)–21 (maximum); melting temperature (*T*_m_) = 52–55–58°C; GC content = 40–50–60%; maximum *T*_m_ difference = 5°C; GC clamp = 1; maximum poly *x* = 4. Forty‐eight primer pairs were chosen to sample the range of repeat types and lengths and product sizes. An M13 tag (Boutin‐Ganache et al., 2001) was added to the 5′ end of the forward primer (CACGACGTTGTAAAACGAC) and a PIG‐tail was added to the 5′ end of the reverse primer (GTTTCTT) to promote non‐template (A) addition (Brownstein et al., 1996).

Primers were tested initially on seven individuals from a range of named species and one tag‐named entity from New Zealand (*C*. “rimutaka,” *C. confusus* Lehnebach, *C. hypogaeus*,* C. macranthus* (Hook. f.) Rchb. f., *C. obscurus*,* C. trilobus*, and *C. walliae* Lehnebach; Appendix 1). DNA was extracted from silica‐dried leaf tissue using a modified cetyltrimethylammonium bromide (CTAB) protocol (Doyle and Doyle, 1987). The 10‐μL PCR cocktail contained 5–50 ng of DNA, 0.02 μM of forward primer, 0.45 μM of reverse primer, 0.45 μM of M13 primer (labeled with FAM, NED, or VIC), 1.5 mM of MgCl_2_, 1× buffer BD (Solis BioDyne, Tartu, Estonia), 250 μM of each dNTP, and 0.5 units of Firepol *Taq* polymerase (Solis BioDyne). The PCR cycling program had an initial denaturation of 95°C for 3 min; 35 cycles of 95°C for 30 s, 53°C for 40 s, and 72°C for 1 min; and a final extension at 72°C for 10 min. PCR products (0.14–1.25 μL) for two to three loci of distinguishable sizes and labeled with different fluorophores were co‐loaded and added to 9 μL of Hi‐Di formamide (Applied Biosystems, Carlsbad, California, USA) and 1 μL of CASS ladder (Symonds and Lloyd, 2004) for subsequent fragment sizing on an ABI 3730 Genetic Analyzer (Applied Biosystems) (Massey Genome Service at Massey University, Palmerston North, New Zealand). Alleles were visualized and scored using GeneMapper version 3.7 (Applied Biosystems).

Of the 48 primer pairs trialed, seven did not amplify, three were unscorable, four were monomorphic, 31 were polymorphic within an individual, and three were polymorphic among species. Twenty‐two loci (Table 1) successfully amplified across all taxa. The number of alleles per locus ranged from one to two in the *C. obscurus* sample and from two to eight in the six other taxa. From these, the 12 most polymorphic loci were used for preliminary population genetic analyses on three populations of *C. obscurus*, one population of *C*. “rimutaka,” and two populations of *C. trilobus* (Table 2, Appendix 1); DNA extraction and locus amplification were as described above. We aimed to sample 15–20 individuals per population, but because of the small and precarious nature of the *C. obscurus* populations, only five individuals per population were included of that species. The total number of alleles, observed heterozygosity (*H*_o_), and expected heterozygosity (*H*_e_) were determined using GenAlEx 6.501 (Peakall and Smouse, 2006). Deviation from Hardy–Weinberg equilibrium (HWE) was determined using the Markov chain method provided by Web version 4.2 of GENEPOP software (Rousset, 2008). The number of alleles ranged from 3–22 (average of 8.8) per locus, *H*_o_ from 0–1 (average of 0.452), and *H*_e_ from 0–0.859 (average of 0.390) (Table 2). All loci except Corybas‐19 deviated significantly from HWE in at least one population. These deviations were usually a lower than expected *H*_o_, suggesting population substructure due to inbreeding or clonality, both of which should be examined more closely in future studies to inform conservation efforts. The average genetic diversity among populations (*F*_ST_) of 0.439 shows the markers are detecting substantial population structure in *Corybas* (Hartl and Clark, 1997).

**Table 1:** Characteristics of 22 microsatellite loci developed for New Zealand *Corybas*

Locus^a^	Primer sequences (5′–3′)	Repeat motif	*C. obscurus*		Other *Corybas* b		GenBank accession no.
			*A*	Allele size range (bp)	*A*	Allele size range (bp)	
Corybas‐05	F: CTCTCTGCACCTTTGGATC	(AG)_6_	1	333	3	331–335	MF076670
	R: TCCCAAGACAAGACTTGAAG						
Corybas‐07	F: CCCAGTGACCAAAGTAGAG	(AG)_7_	1	334	3	332–336	MF076671
	R: TCATGAAGCTTGCATTTGC						
Corybas‐09	F: GCAACTTGTGTCGATTGTC	(AT)_6_	1	372	2	369–372	MF076672
	R: GTCCACTTAGTCCACATGG						
Corybas‐12	F: TCACAACTGGAACTGAACC	(AAG)_9_	2	311–320	4	311–320	MF076673
	R: AGCCCAAACCATCAATCTC						
Corybas‐16	F: ACTCATAGGCCTTAGTGTTG	(AT)_8_	1	269	4	269–275	MF076674
	R: AGAACATCAAACAATGCACG						
Corybas‐18	F: AAATGACAGTGAAGGCCAG	(AAG)_6_	2	298–301	2	298–301	MF076675
	R: TCAAATGTGATGGGCTGAG						
Corybas‐19	F: GTTGGCCCATCAAATATGC	(AT)_6_	1	267	3	251–267	MF076676
	R: TCCTCAATCATGCATGTCC						
Corybas‐22	F: AGATTGCGATGCTGGTTAG	(AG)_7_	2	209–211	2	209–211	MF076677
	R: GAAGACTTCCCTCATCTGC						
Corybas‐23	F: ACAATGGATCCCAAAATCG	(AG)_6_	2	342–354	2	352–354	MF076678
	R: TGCATGGTAGATCAGATGC						
Corybas‐24	F: CCTTTGGAGTTCCCTTGAG	(AT)_6_	1	247	4	245–269	MF076679
	R: CTAATGAAGTGCCTGAGGG						
Corybas‐27	F: TTGCGACACTATGGTAAGC	(AG)_6_	1	240	3	238–242	MF076680
	R: TGACAGGTATGGAAGGTCC						
Corybas‐28	F: GGGATGTGCGTGTATTTTG	(AT)_9_	1	198	3	196–203	MF076681
	R: CAGGTTAAGGCCAGATTCC						
Corybas‐32	F: TTGCCAAGGGGTTTAAGTC	(AT)_9_	1	162	7	162–212	MF076682
	R: ATAAAAGATCATGCACGGC						
Corybas‐33	F: TCACGCTCGCTAATAAGTG	(AG)_8_	2	164–166	2	164–166	MF076683
	R: AAATCCTTCACCAGTCAGG						
Corybas‐36	F: AGCCCTTCACTATTGTCAG	(AG)_10_	1	152	4	146–152	MF076684
	R: TGAACCTTATGCAATCTCC						
Corybas‐41	F: TATGTTTGGGGTCTTTCGC	(AG)_7_	2	155–157	2	155–157	MF076685
	R: CGGGAATTCCTCTCATTCC						
Corybas‐42	F: AAGGTACTCTGTGAGGTCG	(AG)_8_	1	333	3	325–345	MF076686
	R: TCTTTGCTAGTTGAAGGCC						
Corybas‐44	F: CTGCAGATTTGTTGTGCTG	(AT)_15_	2	214–218	6	218–248	MF076687
	R: TTCCGAATCACGATGACTG						
Corybas‐45	F: CATTTTCGGCACAACTCTC	(AG)_9_	2	264–266	8	262–284	MF076688
	R: ACCAGCAGTAATACACAGC						
Corybas‐46	F: TGAATTTTAGCATGCGCAG	(AT)_6_	1	187	2	184–187	MF076689
	R: ATGCCACTATCACTGTTGC						
Corybas‐47	F: GTATGTCGATAGGCCTTGG	(AAC)_10_	2	332–346	8	317–349	MF076690
	R: CCACTAGGGACAAGTTTGG						
Corybas‐48	F: ACACTTCAAATAGGCATAGG	(ATC)_9_	2	178–187	8	178–247	MF076691
	R: CGATAAGGAGTGCAATTGC						

Note

*A* = number of alleles.

a Annealing temperature for all loci was 53°C.

b *Corybas confusus*,* C. hypogaeus*,* C. macranthus*,* C. obscurus*,* C*. “rimutaka,” *C. trilobus*, and *C. walliae*.

**Table 2:** Genetic properties of the 12 newly developed microsatellite loci for three populations of *Corybas obscurus*, one population of *C*. “rimutaka,” and two populations of *C. trilobus*.^a^

Locus	*Corybas obscurus*												*Corybas* “rimutaka”				*Corybas trilobus*								Total (*n* = 73)
	Site 1 (*n =* 5)				Site 3 (*n =* 5)				Site 4 (*n =* 5)				East Harbour Regional Park (*n* = 21)				Sutherland's Bush (*n* = 21)				Gordon Park (*n* = 16)				
	*A*	Allele size range (bp)	*H*_o_ b	*H*_e_	*A*	Allele size range (bp)	*H*_o_ b	*H*_e_	*A*	Allele size range (bp)	*H*_o_ b	*H*_e_	*A*	Allele size range (bp)	*H*_o_ b	*H*_e_	*A*	Allele size range (bp)	*H*_o_ b	*H*_e_	*A*	Allele size range (bp)	*H*_o_ b	*H*_e_	*A*_T_
Corybas‐07	1	334	0.000	0.000	1	334	0.000	0.000	1	334	0.000	0.000	3	305–334	0.429	0.407	2	332–334	0.000***	0.308	2	332–334	0.000***	0.492	3
Corybas‐12	1	312	0.000	0.000	2	311–314	1.000	0.500	2	311–322	1.000	0.500	2	311–314	0.190	0.245	5	314–334	0.524**	0.573	3	314–322	0.625*	0.635	9
Corybas‐16	2	283–313	1.000	0.500	2	267–271	1.000	0.500	2	313–321	1.000	0.500	6	267–277	0.750	0.705	8	269–285	0.857***	0.845	5	269–283	0.333***	0.638	13
Corybas‐19	1	267	0.000	0.000	1	267	0.000	0.000	2	267–275	1.000	0.500	2	265–267	0.048	0.046	2	251–265	0.095	0.091	2	265–267	0.071	0.191	4
Corybas‐23	2	342–354	1.000	0.500	1	352	0.000	0.000	2	352–354	1.000	0.500	2	319–352	0.053*	0.229	1	352	0.000	0.000	2	352–354	0.063	0.061	4
Corybas‐24	2	246–247	1.000	0.500	2	246–247	1.000	0.500	1	247	0.000	0.000	3	245–253	0.316*	0.578	4	245–257	0.400***	0.747	3	245–251	0.091**	0.368	6
Corybas‐28	1	198	0.000	0.000	1	196	0.000	0.000	1	196	0.000	0.000	2	196–203	0.000*	0.095	2	203–205	0.190	0.172	2	199–203	0.438	0.342	5
Corybas‐32	1	162	0.000	0.000	1	172	0.000	0.000	2	184–188	1.000	0.500	8	164–184	0.400***	0.778	6	164–180	0.619***	0.772	5	164–180	0.200***	0.669	11
Corybas‐36	1	152	0.000	0.000	2	152–158	1.000	0.500	1	152	0.000	0.000	4	146–152	0.450*	0.694	4	146–152	0.571***	0.677	3	148–152	0.625	0.646	5
Corybas‐44	2	214–230	1.000	0.500	1	218	0.000	0.000	1	228	0.000	0.000	8	214–232	0.857	0.787	8	218–234	0.905***	0.854	8	218–238	0.750***	0.809	12
Corybas‐45	2	264–280	1.000	0.500	1	264	0.000	0.000	2	258–264	1.000	0.500	7	262–284	0.619	0.791	6	262–282	0.381***	0.762	5	262–280	0.250***	0.641	12
Corybas‐48	2	181–187	1.000	0.500	2	181–202	1.000	0.500	2	181–205	1.000	0.500	13	178–259	0.750	0.859	8	184–226	0.750***	0.859	6	184–211	1.000***	0.715	22
Average	1.5		0.500	0.250	1.4		0.417	0.208	1.6		0.583	0.292	5		0.405	0.518	4.7		0.441	0.555	3.8		0.371	0.517	8.83

Note

*A* = number of alleles; *A*_T_ = total number of alleles; *H*_e_ = expected heterozygosity; *H*_o_ = observed heterozygosity; *n* = number of sampled individuals.

^a^Locality and voucher information are provided in Appendix 1.

^b^Significance of deviation from Hardy–Weinberg equilibrium: *P < 0.05, **P < 0.01, ***P < 0.001.

To test the transferability of the markers for use in species delimitation, 38 individuals from both the North and South Island were chosen, representing four species and one tag‐named entity (*C. hypogaeus*,* C. obscurus*,* C. vitreus* Lehnebach, *C. walliae*, and *C*. “rimutaka”; 3–16 individuals per taxon; Appendix 1). For these, we amplified the 12 novel microsatellite markers and genotyped them as described above. GenAlEx 6.501 was used to determine the percentage of successful amplifications per locus and *F*_ST_. Amplification success rate was 95.8% on average, ranging from 81.58% to 100% amplification across all taxa (Table 3). Alleles ranged from 2.8–5 per species, with an average of 8.4 across all loci and species.

**Table 3:** Allelic properties and amplification results of microsatellite loci isolated from *Corybas obscurus* and tested across four additional *Corybas* species.^a^

Locus	*C. “*rimutaka*”* (*n* = 7)		*C. walliae* (*n* = 7)		*C. obscurus* (*n* = 16)		*C. hypogaeus* (*n* = 5)		*C. vitreus* (*n* = 3)		*A*_T_	Successful amplification (%)
	*A*	Allele size range (bp)	*A*	Allele size range (bp)	*A*	Allele size range (bp)	*A*	Allele size range (bp)	*A*	Allele size range (bp)		
Corybas‐07	3	332–336	1	334	2	334–336	3	332–334	1	332	3	100
Corybas‐12	4	311–320	7	311–334	4	311–322	5	311–334	3	311–317	8	100
Corybas‐16	4	267–303	6	265–323	6	267–321	5	267–283	4	267–291	13	95
Corybas‐19	2	265–267	2	265–267	3	265–275	2	265–267	3	265–269	4	95
Corybas‐23	2	342–352	3	342–354	3	342–354	1	352	1	352	3	95
Corybas‐24	4	245–247	2	245–247	2	246‐247	4	245–261	2	247–251	5	92
Corybas‐28	1	203	3	196–203	2	196–198	2	203–205	2	196–203	4	97
Corybas‐32	4	164–174	9	160–184	5	162–188	1	162	1	180	11	82
Corybas‐36	5	146–156	7	146–166	2	152–158	3	148–152	3	146–150	8	97
Corybas‐44	4	214–226	5	216–240	4	214–230	3	218–226	6	224–242	13	100
Corybas‐45	7	262–284	7	262–274	4	258–280	1	262	3	266–272	11	97
Corybas‐48	8	178–256	8	178–220	6	178–205	7	184–214	5	184–208	18	100
Average	4		5		3.6		3.1		2.8		8.4	95.83

Note

*A* = number of alleles; *A*_T_ = total number of alleles; *n* = number of sampled individuals.

a Locality and voucher information are provided in Appendix 1.

## CONCLUSIONS

We developed 22 polymorphic microsatellite markers from *C. obscurus* that amplified to varying degrees in seven congeneric species and one undescribed entity. Twelve markers amplified reliably across seven species and were further tested on multiple populations and species to test their amplification across species and potential utility for population genetics. Due to the high success rate of amplification and the number of polymorphic loci, these markers will be informative for population genetics, mating system analysis, species delimitation, and determining the extent of hybridization within populations of mixed species. As such, these markers will facilitate the development of a conservation strategy for these species in New Zealand, as well as Australia.

## DATA ACCESSIBILITY

The raw data were deposited in the National Center for Biotechnology Information (NCBI) Sequence Read Archive (accession SRP150798); primer sequences were uploaded to GenBank, and accession numbers are provided in Table 1.

## Appendix 1 Voucher and locality information for the New Zealand *Corybas* samples used in this study.^a^

**Table:**

Species/form	*n*	Voucher no.	Population
*Corybas* “rimutaka”	1	WELT‐SP105870^c^	North Island, Rimutaka Forest Park
*Corybas* “rimutaka”	2	WELT‐SP105871^e^	North Island, Rimutaka Forest Park
*Corybas* “rimutaka”	21	WELT‐SP104172^d^	North Island, Wellington, East Harbour Regional Park
*Corybas* “rimutaka”	3	WELT SP105940^e^	North Island, Wellington, QEII Covenant, Eastborne
*Corybas* “rimutaka”	1	WELT‐SP104159^e^	South Island, Mt. Cook National Park
*Corybas* “rimutaka”	1	WELT‐SP105872^e^	South Island, Nelson Lakes, Six Mile Creek
*Corybas confusus* Lehnebach	1	WELT‐SP104160^c^	South Island, Mt. Cook National Park
*Corybas hypogaeus* (Colenso) Lehnebach	1	WELT‐SP104185^e^	North Island, Hawke's Bay, Boundary Stream Mainland Island
*Corybas hypogaeus*	1	WELT‐SP104417^e^	North Island, Te Urewera National Park
*Corybas hypogaeus*	1	WELT‐SP104177^e^	North Island, Ohakune, Tongariro National Park
*Corybas hypogaeus*	1	WELT‐SP104416^c^	South Island, Nelson Lakes National Park
*Corybas hypogaeus*	1	WELT‐SP105873^e^	South Island, Nelson Lakes National Park
*Corybas hypogaeus*	1	WELT‐SP105874^e^	South Island, Nelson Lakes National Park, Rainbow Ski field
*Corybas macranthus* (Hook. f.) Rchb. f.	1	WELT‐SP105875^c^	South Island, Nelson Lakes National Park
*Corybas obscurus* Lehnebach	1	WELT‐SP104152^b^^,^ e	South Island, Nelson Lakes National Park
*Corybas obscurus*	1	WELT‐SP104152^c^	South Island, Nelson Lakes National Park
*Corybas obscurus*	5	WELT‐SP104152^d^^,^ e	South Island, Nelson Lakes National Park, Site 1
*Corybas obscurus*	5	WELT‐SP106571^d^^,^ e	South Island, Nelson Lakes National Park, Site 3
*Corybas obscurus*	5	WELT‐SP106570^d^^,^ e	South Island, Nelson Lakes National Park, Site 4
*Corybas trilobus* (Hook. f.) Rchb. f.	21	WELT‐SP104195^c^^,^ d	North Island, Turakina Valley, Sutherland's Bush
*Corybas trilobus*	16	WELT‐SP104181^d^	North Island, Whanganui, Gordon Park Scenic Reserve
*Corybas vitreus* Lehnebach	1	WELT‐SP105876^e^	South Island, Richmond Forest Park, Inwood Lookout
*Corybas vitreus*	1	WELT‐SP107154^e^	South Island, Glenorchy, Glacier Burn track
*Corybas vitreus*	1	WELT‐SP107155^e^	South Island, Nelson Lakes, Rainbow Station
*Corybas walliae* Lehnebach	2	WELT‐SP104410^e^	North Island, Egmont National Park
*Corybas walliae*	1	WELT‐SP104178^e^	North Island, Ruahine Ranges Forest Park
*Corybas walliae*	1	WELT‐SP104175^e^	North Island, Tongariro National Park
*Corybas walliae*	1	WELT‐SP105877^e^	South Island, Glen Hope Scenic Reserve
*Corybas walliae*	2	WELT‐SP104391^e^	South Island, Kahurangi National Park
*Corybas walliae*	1	WELT‐SP104151^c^	South Island, Nelson Lakes National Park

Note

*n* = number of individuals genotyped.

a One voucher was collected from each population used; vouchers are deposited in WELT. Latitude and longitude are not provided to suppress detailed locality information.

b Individual used for library construction.

c Individuals used for tests of amplification and polymorphism.

d Individuals used for population analyses.

e Individuals used to test markers on different forms and a wider range of sampling across the North and South Islands.
